# Diuretic, Sedative and Diaphoretic Pill

**Published:** 1888-09

**Authors:** J. B. Johnson

**Affiliations:** Washington, D. C.


### DIURETIC, SEDATIVE AND DIAPHORETIC PILL.

By J. B. JOHNSON, M. D., WASHINGTON, D. C.

I have been using the following pill for many years as a substitute for Dover's powder, and in all cases where a diuretic, sedative and diaphoretic are indicated, have found this combination most efficacious:

℞. Sulp. morphia ........................ grs. ijss,
Pulv. opii ........................ grs. xv,
Pulv. digitalis ........................ grs. xv,
Pulv. scillæ ........................ grs. xv,
Fld. ext. hyoscyam ........................ gtts. xxij.

M. Make pills No. 20. One every three hours.

In the ordinary practice of medicine, many cases are met which require, not only a control of pain, nervous irritability and rapid circulation, but also an increase of the secretions of both skin and kidneys. These indications are served in a most happy manner by the administration of this pill. I use these pills in order to control the restlessness of pneumonia, the troublesome cough of bronchitis, the pain caused by pleurisy and rheumatism, and to quiet the inflammation and pain which accompany the acute or chronic conditions of diseases of the organs of the female pelvis. I always have these pills with me, and use them freely in all cases where it is necessary to stop pain, quiet the heart, subdue the restlessness, soothe the nervous system, and to increase the action of the skin and kidneys. When opium or its preparations are given alone, their action is to stop all the secretions except that of the skin; but this objection seems to be obviated by the addition of digitalis and squill; and the harmonious action of these pills, when wisely given, is most satisfactory to me and agreeable to my patient.—*Southern Clinic.*

